# Progress and challenges in seasonal influenza vaccination across 54 countries and areas in the WHO European Region, 2008/09–2022/23: a repeated cross-sectional ecological study

**DOI:** 10.1016/j.lanepe.2026.101681

**Published:** 2026-04-19

**Authors:** Margaux Marie Isabelle Meslé, Marc-Alain Widdowson, Pernille Jorgensen

**Affiliations:** a World Health Organization, Regional Office for Europe, Marmorvej 51, UN City, Copenhagen, 2100, Denmark

**Keywords:** Seasonal influenza vaccination, Vaccination policy, Vaccine coverage, Pandemic preparedness, WHO European Region

## Abstract

**Background:**

Seasonal influenza causes up to 5 million severe cases and 650,000 deaths annually. Vaccination is recommended for high-risk groups and health workers, yet uptake remains low globally. This paper analyses the evolution of seasonal influenza vaccination programmes in the WHO European Region’s 54 countries and areas (CA) from 2008/09 to 2022/23 to guide future policy and investment.

**Methods:**

We conducted a descriptive, repeated cross-sectional ecological study using national data reported through WHO-UNICEF’s Joint Reporting Form on Immunization and reports published by the European Centre for Disease Prevention and Control to analyse influenza vaccine doses, vaccine types, recommendations, coverage, and payment schemes. Trends in doses distributed per capita and older-adult vaccination coverage over 15 seasons were assessed across World Bank income groups.

**Findings:**

Since 2008/09, the number of influenza vaccine doses distributed has doubled, driven mainly by increases in upper- (3-fold) and lower-middle-income (8-fold) CA. Yet, by 2022/23, dose availability per 1000 population remained substantially greater in high-income than lower-middle-income settings (145.7 versus 38.5). Recommendations expanded over time to additional target populations, but coverage monitoring remains limited, with fewer than 50% of CA reporting data for key groups, such as health workers. In 2022/23, median coverage among older adults was 55%, and only four CA met the World Health Assembly 75% coverage target.

**Interpretation:**

Considerable progress has been made in influenza vaccination across the WHO European Region, with an increasing number of CA recommending vaccination to key target groups. Nonetheless, coverage among priority groups is low, and vaccine supplies vary widely across income levels. Continued investment in national programmes is needed, especially in middle-income CA to keep increasing coverage among high-risk groups.

**Funding:**

This work was supported by the Pandemic Influenza Preparedness Framework Partnership Contribution.

**Research in context**

**Evidence before this study**

We searched PubMed for multi-country publications on seasonal influenza vaccination recommendations and vaccination coverage in the WHO European Region. We used combinations of the following keywords/terms: ‘influenza vaccination’/‘influenza immunization’, ‘recommendation’/‘guideline’/‘policy’ and ‘coverage’/‘uptake’, together with geographical terms (‘Europe’, ‘European Region’, ‘EU’, ‘EEA’) and target-group terms (older adults/elderly, health-care workers, pregnant women/pregnancy, children/paediatric, chronic conditions/comorbidity). The search strategy excluded clinical trials and intervention studies and was limited to publications from 1 January 2020 to 1 December 2025. Most published studies reported vaccination coverage for single countries or single target groups, often for only one or a few seasons, and many relied on already published data. Several studies reported coverage data only for subpopulations within target groups or for selected geographical locations rather than national coverage estimates for the entire target population. Only one study reported on recommendations and influenza vaccination coverage for the entire WHO European Region, however, these were aggregated at the regional level and largely limited to 2022. Overall, we found that contemporary, harmonized, multi-season (time-series) data on influenza vaccination coverage and recommendations across the whole WHO European Region remain limited.

**Added value of this study**

This paper provides the first comprehensive analysis of seasonal influenza vaccination programmes across all countries and areas of the WHO European Region since the COVID-19 pandemic. Over 15 seasons (2008/09–2022/23, inclusive), influenza vaccine distribution increased substantially, and recommendations for priority groups expanded. Nonetheless, major gaps in vaccination coverage among target groups remain, highlighting areas where programme implementation falls short. Our findings provide region-wide evidence to inform future investment and policy development to strengthen influenza vaccination programmes.

**Implications of all the available evidence**

Our findings show that current influenza vaccination strategies remain insufficient to protect populations at risk across the WHO European Region. Substantial gaps in vaccination coverage persist across all income groups, most pronounced in lower-resourced settings, despite widespread vaccination policies. Accelerated global efforts are needed to improve access to affordable and effective influenza vaccines, alongside national initiatives to identify and address barriers to vaccination.

## Introduction

Seasonal influenza is a major global health issue, responsible for an estimated 3–5 million severe cases and up to 650,000 respiratory deaths each year, placing a substantial burden on healthcare systems.^1^ Beyond respiratory illness, influenza infection has been linked to an increased risk of cardiovascular events and related mortality.^2^ Moreover, in Europe, seasonal influenza has been identified as the infectious disease with the highest impact on population health as measured in disability-adjusted life years (DALYs)^3^ and is associated with significant excess mortality in some winter seasons.^4^

Older adults, young children, pregnant women, and individuals with comorbidities are at higher risk of severe influenza-related illness, hospitalization, and death.^5^ Among these, individuals aged ≥ 65 years experience the largest mortality burden accounting for almost 70% of all influenza-related respiratory deaths.^6^ Accordingly, the World Health Organization’s (WHO) Strategic Advisory Group of Experts (SAGE) on Immunization classifies these four populations as priority target groups for vaccination.^7^ Furthermore, a fifth group, health workers, is recommended for vaccination to protect staff and reduce the risk of transmitting influenza to vulnerable patients. In addition, WHO notes that other groups to be considered for vaccination include people at high risk of severe influenza living in congregate settings (e.g., prisons, refugee camps, and group homes) and emphasises that programmes should promote vaccination among disadvantaged and Indigenous populations with a high burden of disease.

Seasonal influenza vaccination is also increasingly being recognized as a cornerstone of pandemic preparedness.^8^^,^^9^ Experience from both the 2009 A(H1N1) and the COVID-19 pandemics showed that countries with established seasonal influenza vaccination programmes deployed pandemic vaccines more rapidly than those with limited or no programmes, by leveraging existing infrastructure, supply chains and delivery systems.^8^^,^^10^, ^11^, ^12^ The significance of seasonal influenza vaccination for pandemic preparedness is also reflected in WHO’s Pandemic Influenza Preparedness (PIP) Framework adopted by all Member States at the 64th World Health Assembly (WHA) (2011).^13^

Given the importance for both disease prevention and pandemic preparedness, understanding how seasonal influenza vaccination programmes in the WHO European Region (WHO/Europe) have developed over time, including through the COVID-19 pandemic, is essential for supporting evidence-based policies and guiding national immunization resources. Building on previously published data, this paper provides an updated overview of the status and evolution of seasonal influenza vaccination programmes in WHO/Europe from 2008/09 to 2022/23. Using 15 years of data, we examined temporal changes in and the current status of vaccine recommendations, availability, and coverage, including progress toward the WHA target of 75% coverage among older adults.

## Methods

### Data sources

We conducted a descriptive, repeated cross-sectional ecological study using officially reported influenza vaccine programme data for the period 2008/09 to 2022/23 from the 53 WHO/Europe Member States and Kosovo (in accordance with United Nations Security Council resolution 1244 (1999)), hereafter collectively referred to as countries and areas (CA). The unit of analysis was the CA-season (country/area-season). Data were obtained from three sources: i) published reports on seasonal influenza vaccination programmes among European Union and European Economic Area (EU/EEA) Member States conducted by the European Centre for Disease Prevention and Control (ECDC) and the Vaccine European New Integrated Collaboration Effort (VENICE) project covering the 2008/09–2014/15 seasons^14^; ii) a survey conducted by WHO/Europe for the same period^15^; and iii) the global WHO/UNICEF Joint Reporting Form on Immunization (JRF) survey covering all CA in WHO/Europe for the period 2015/16 to 2022/23.^16^ The number of CA that were invited to participate in the surveys increased over time, from 50 in 2008/09 to 54 from 2019/20 onwards. The scope of information collected also broadened during this period, from basic programme data on doses procured, coverage and recommendations to additional programmatic aspects (Supplementary Table S1). All CA responding to the JRF, WHO/Europe, or VENICE surveys were included. Because availability of each indicator varied by data source and season, each indicator was analysed using all available (non-missing) CA-season observations (i.e., analyses were indicator-specific and did not require complete reporting across all indicators). Additional completeness criteria were applied for temporal trend analyses (see Statistical analysis). No formal sample size calculation was undertaken as this study is a descriptive analysis of routinely collected CA-reported data.

### Variables

We extracted information on seasonal influenza vaccination programme indicators, including vaccine doses distributed (or procured where distribution data were unavailable), target-group-specific recommendations, coverage, and payment (e.g., free, copayment), as well as vaccine types (trivalent, quadrivalent, live attenuated influenza vaccine (LAIV), high-dose, and adjuvanted). The target group categories for which data were collected included children (<18 years, without comorbidities), persons with comorbidities (reported separately for children <18 years and adults ≥ 18 years), older adults (above a nationally defined age threshold), pregnant women, health workers, residents living in long-term care facilities, and “other” populations. Details on target group recommendations (e.g., age thresholds and other characteristics) were defined by each CA. For the purposes of this study, a national influenza vaccination programme was defined as having recommendations in place for at least one of the five priority groups identified by SAGE.

The primary outcomes were vaccine doses distributed (or procured) per capita and influenza vaccination coverage by target group. Doses per capita were calculated using mid-year population estimates from the United Nations Population Division^17^ for the corresponding year and reported as doses per 1000 population. Vaccination coverage was defined as the proportion (%) of the nationally defined target population vaccinated against influenza in a given season, as reported by each CA (administrative or survey-based). Secondary indicators included presence of recommendations by target group and payment policy, and vaccine type availability, which were summarised descriptively across CA and seasons.

### Statistical analysis

Descriptive analyses were conducted to assess changes in seasonal influenza vaccination programme indicators across CA in WHO/Europe between 2008/09 and 2022/23, accounting for differences in the number of CA reporting and indicators included in the different surveys across the years (see Supplementary Table S1). Indicators were summarised by season using counts and percentages for categorical indicators and medians (interquartile ranges, IQR) for continuous indicators. Analyses were conducted at the CA-season level. Regional summaries were calculated as unweighted medians of reported CA-level coverage estimates and doses procured, and do not represent population-weighted regional estimates. Pooled regional estimates and confidence intervals were not calculated. Analyses of temporal trends were limited to indicators with data available for at least 12 of the 15 seasons, representing ≥80% completeness, and reported by more than 50% of CA across the Region. Based on these criteria, two indicators - vaccine doses distributed per capita and coverage among older adults - met the inclusion criteria for temporal trend analysis. For these trend analyses, where one season or two consecutive seasons were missing, we imputed the value using the average of the preceding and following seasons. For the first (2008/09) and last (2022/23) seasons, any missing data were imputed using the subsequent (2009/10) or preceding (2021/22) season, respectively. CA with gaps of three consecutive seasons were excluded from analysis. Applying these criteria, 33 CA were included in the temporal analysis on coverage among older adults and 28 CA in the analysis of doses distributed per capita. Vaccine doses distributed (or procured) per capita and older-adult coverage were summarised by season and stratified by CA income group, using the World Bank^18^ income classifications (low-middle (LMIC), upper-middle (UMIC), and high-income (HIC)) based on Gross National Income (GNI) per capita (US$) (Supplementary Table S2).

To assess whether observed temporal patterns in influenza vaccination coverage among older adults were affected by changes in the CA contributing data over time, we conducted a sensitivity analysis restricted to CA with coverage data available for all seasons (2008/09 to 2022/23) (n = 24). We compared season-specific median coverage across CA from this restricted dataset with the main analysis, which included CA with ≥80% complete data (with imputation for one missing season or two consecutive missing seasons as described above).

All data analysis was performed using R version 4.3.1, with packages tidyverse and ggplot2.^19^

The study is reported according to the “STrengthening the Reporting of OBservational studies in Epidemiology” (STROBE) guidelines for observational studies.

### Ethical approval

This study used routinely reported, aggregated CA-level data that are publicly available and contained no individual-level or identifiable information. Ethics committee review and informed consent were not required.

### Role of the funding source

The funder had no role in study design, data collection, analysis, interpretation, or writing of this manuscript.

## Results

Response rates from CA to the annual surveys on seasonal influenza vaccination remained consistently high over the 15-year study period; the lowest was 91% (48 of 53) in 2018/19 and the highest was 98% (53 of 54) in 2022/23 (Supplementary Fig. S1).

### Influenza vaccine volumes and product characteristics

**Fig. 1:**
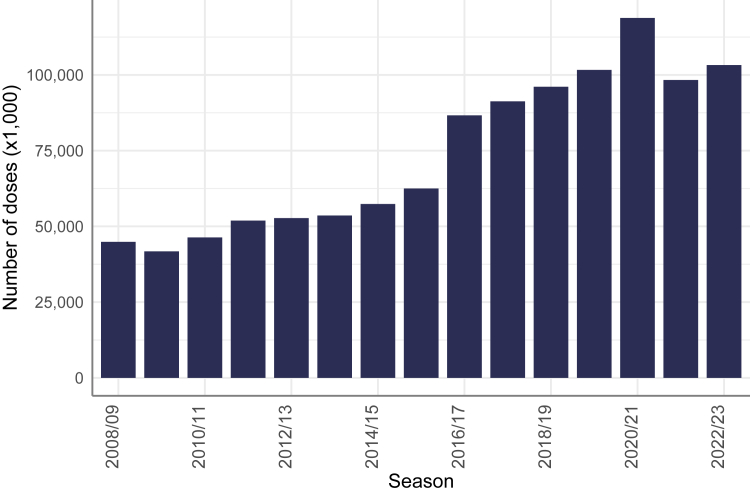
Number of doses distributed or procured per season among countries and areas that reported at least 12 seasons of data (n = 28).^b^

Among the 28 CA that reported data on influenza vaccine doses distributed (or procured) for 12 or more seasons, the total number of doses distributed increased from 45 million in 2008/09 to 103 million in 2022/23, peaking at 119 million doses in 2020/21 (Fig. 1). The rise in number of doses distributed as of 2016/17 was largely driven by UMICs.

**Fig. 2:**
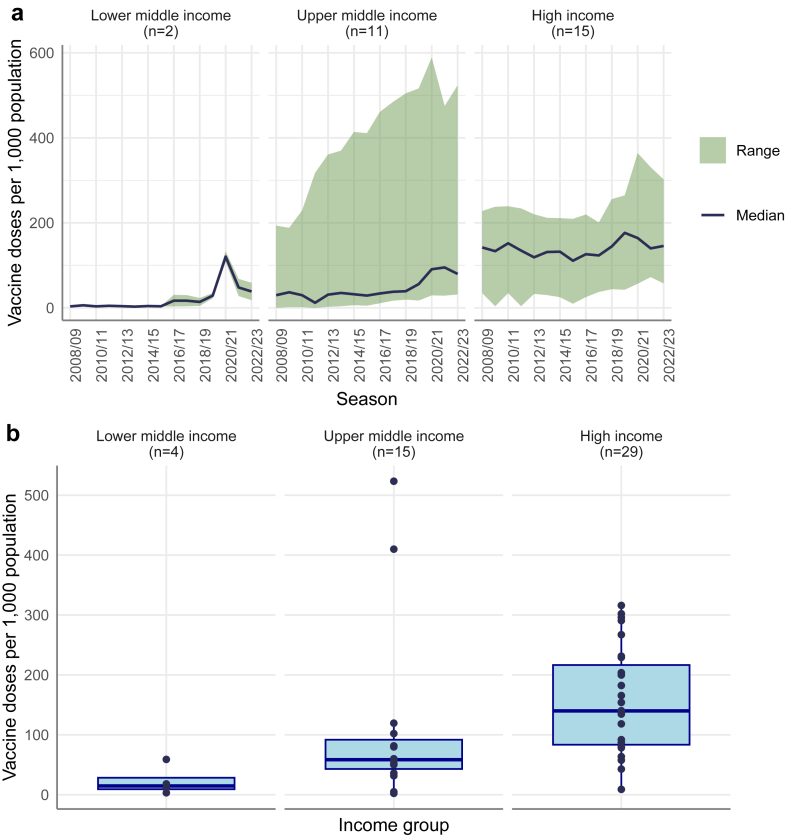
a) Number of seasonal influenza vaccine doses per 1000 population distributed or procured (minimum, mean, median and maximum) by income group and season, among countries and areas reporting at least 12 seasons of data. b) Boxplot showing vaccine doses distributed per 1000 population in 2022/23 stratified by income group.

There were notable differences in CA-level dose availability by income group over the 15-year period with HICs consistently distributing a higher number of influenza vaccines per capita than UMICs and LMICs (Fig. 2a). From 2008/09 to 2022/23, however, the CA-level median number of doses per 1000 population remained relatively stable in HICs from 142.1 to 145.7, while doses in UMICs and LMICs increased 2.7-fold (29.8 to 79.8) and 8.4-fold (3.4 to 38.5), respectively, among the 28 CA reporting data for ≥12 years. All income groups reported a temporary increase in doses in 2020/2021–the first influenza season during the COVID-19 pandemic.

In the most recent season (2022/23), among the 48 CA reporting data, the median number of doses distributed (or procured) per 1000 population was 139.9 in HICs, compared with 58.5 in UMICs and 14.6 in LMICs (Fig. 2b).

**Fig. 3:**
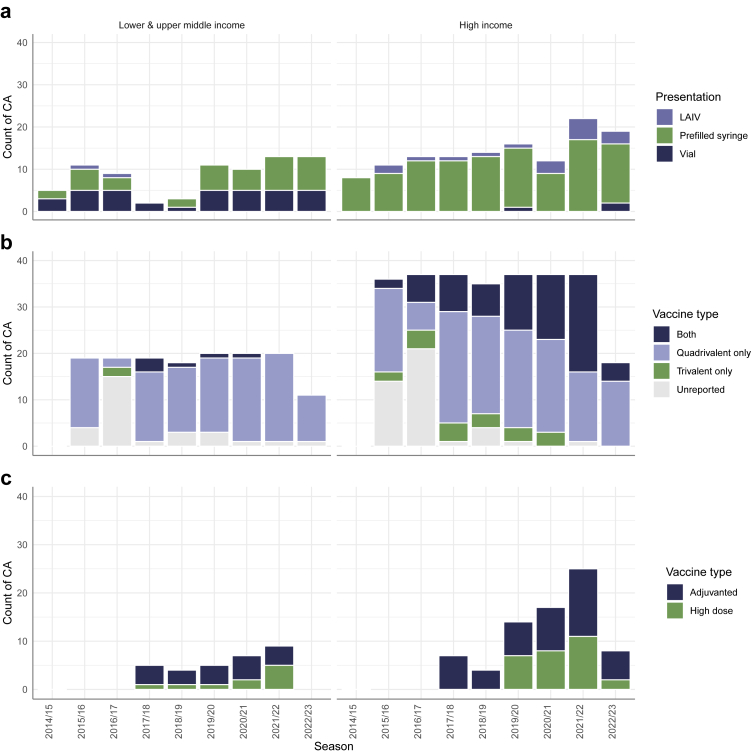
Number of countries and areas reporting: a) vaccine dose presentation; b) use of trivalent, quadrivalent vaccines or both; c) use of adjuvanted or high-dose vaccines, by influenza season and income group.

Reporting completeness for vaccine presentation and formulation varied considerably by year and CA (Fig. 3). Prefilled syringes were the most commonly reported presentation overall, particularly in HICs, whereas in UMICs and LMICs, prefilled syringes and vials (single or multidose) were reported in similar proportions. LAIVs were reported less frequently and were not used in UMICs and LMICs in recent years. Furthermore, data suggested an increasing use of quadrivalent vaccines, gradually replacing trivalent formulations over time during the study period. Use of high-dose and adjuvanted vaccines also appeared to rise, especially in HICs, however, reporting was very inconsistent across years.

### Influenza vaccination recommendations, coverage and cost

**Fig. 4:**
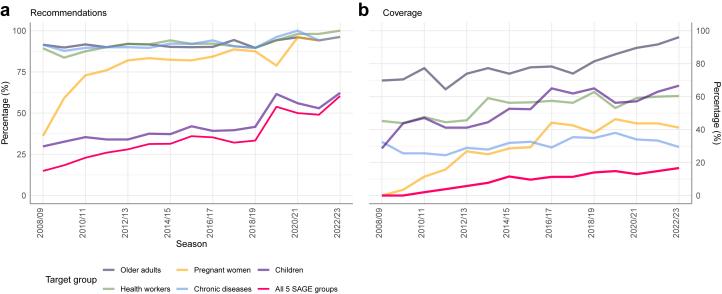
Percentage of countries and areas: a) with seasonal influenza vaccination recommendations; b) reporting seasonal influenza vaccination coverage (among those with recommendations in place), by target group.

**Fig. 5:**
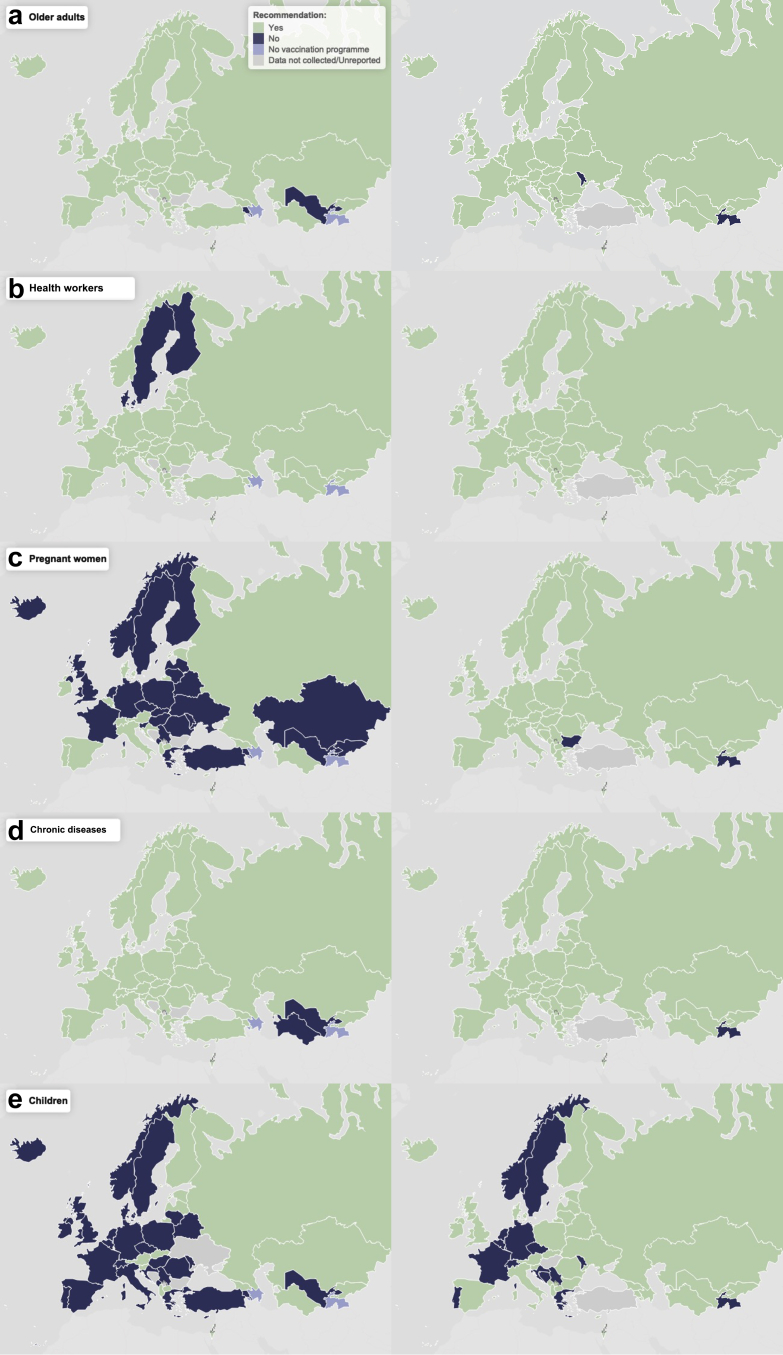
Overview of seasonal influenza vaccination recommendations in the WHO European Region in the 2008/09 (left) and 2022/23 (right) seasons by target group: a) older adults; b) health workers; c) pregnant women; d) persons with chronic underlying conditions; and e) children.^c^

The percentage of CA that reported recommendations for all five SAGE-recommended target groups (older adults, children, pregnant women, populations with chronic conditions, and health workers) increased from 15% (7 of 47) in 2008/09 to 60% (32 of 53) in 2022/23. Of the five target groups, children and pregnant women saw the largest increase in CA introducing recommendations: 54% and 64% increase, respectively (Fig. 4a, Fig. 5).

### Older adults

In 2008/09, 91% (43 of 47) of CA recommended seasonal influenza vaccination for older adults, which by 2022/23 had increased to 96% (51 of 53). In 2022/23, most recommendations specified vaccination from age ≥ 65 years (n = 36 CA, 71%), while 11 CA (22%) recommended vaccination from ≥60 years; the remaining four CA (8%) did not specify an age threshold (Supplementary Fig. S2). Among CA with vaccine recommendations, the proportion reporting coverage data increased from 30 of 43 (70%) in 2008/09 to 49 of 51 (96%) in 2022/23 (Fig. 4b).

**Fig. 6:**
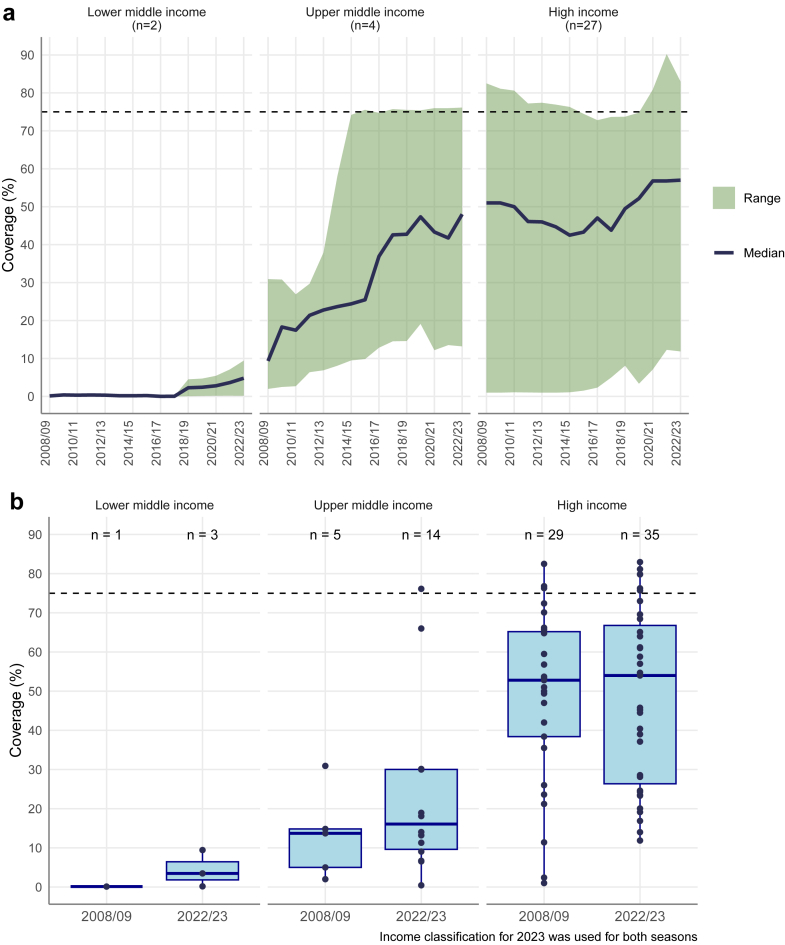
a) Median and range (min-max) of seasonal influenza vaccination coverage among older adults, by income group, for 33 countries and areas with data reported for ≥12 seasons. b) Boxplot of vaccination coverage among older adults in 2008/09 and 2022/23, by income group.^d^

Across the sub-group of CA with ≥12 seasons of data (n = 33), the median CA-level vaccination coverage among older adults increased overall across all three income groups, with the largest absolute increase among UMICs, while the highest median CA-level coverage was among HICs (Fig. 6a). The median CA-level coverage across the Region increased from 49% [IQR: 23%–59%] in 2008/09 to 55% [IQR: 24%–66%] in 2022/23, with country-level values ranging from <1% to >80% across the period. The sensitivity analysis showed a comparable temporal pattern to the main analysis, although median older-adult coverage was consistently higher among CA with complete reporting across all seasons (Supplementary Fig. S4).

When all available coverage data were considered for 2008/09 and 2022/23 (i.e., not restricted to CA with ≥12 seasons of data), HICs also consistently reported the highest median CA-level coverage in both seasons (53% [IQR: 38%–65%] and 54% [IQR: 26%–67%], respectively) (Fig. 6b), and while the median remained largely similar over the 15-year period, fewer CA reported very low coverage in 2022/23. UMICs reported substantially lower CA-level median coverage than HICs (14% [IQR: 5%–15%] in 2008/09 and 16% [IQR: 10%–30%] in 2022/23). The median remained largely stable over time; however, variability increased considerably in 2022/23, reflecting a larger number of CA reporting data, including CA with both very low- and high reported coverage. LMICs had the lowest coverage in both years, with CA-level medians of 0.1% [IQR: 0.1% to 0.1%] and 3.5% [IQR: 1.8% to 6.5%] respectively. Across all income groups, only a few CA met the WHA target of 75% coverage; four CA in 2022/23 (three HICs and one UMIC), compared with two in 2008/09 (both HICs).

Free provision of influenza vaccination for older adults ranged from 84% (n = 38, 2020/21) to 85% (n = 41, 2022/23) among CA reporting payment data for the period 2020/21–2022/23 (Supplementary Fig. S5).

### Health workers

CA with recommendations for health workers increased from 89% (42 of 47) of CA in 2008/09 to 100% (53 of 53) in 2022/23 (Fig. 4). National policies varied, with some CA recommending vaccination for all health workers, while others targeted specific subgroups, e.g., only health workers with patient contact (including medical and non-medical staff), frontline staff, or specific medical specialties, such as paediatricians, family doctors, nurses, medical staff in intensive care units. For those CA recommending vaccination, the proportion reporting vaccination coverage data rose from 45% (19 of 42) in 2008/09 to 60% (32 of 53) in 2022/23 (Fig. 4). Reported coverage ranged from 14% to 98% in 2008/09 and from 4% to 99% in 2022/23.

CA reporting vaccination free of charge for health workers varied from 73% (n = 32, 2020/21) to 79% (n = 38, 2022/23) (Supplementary Fig. S5).

### Pregnant women

Over the study period, national recommendations for influenza vaccination in pregnant women expanded, reaching 96% (51 of 53) in 2022/23, up from 36% (17 of 47) of CA in 2008/09 (Fig. 4). Among the 51 CA with recommendations in 2022/23, 31 provided details on the target group. Most (n = 23, 74%) advised vaccination for all pregnant women regardless of trimester or health status, six (19%) recommended vaccination from the second trimester only, and two (6%) only for those with underlying medical conditions. Coverage reporting for this group increased from none in 2008/09 to 41% (21 of 51) in 2022/23 with values ranging from <1% to 97%.

Where payment information was available, the proportion of CA reporting free influenza vaccination for pregnant women ranged from 85% (n = 35, 2020/21) to 87% (n = 41, 2022/23) (Supplementary Fig. S5).

### Persons with chronic underlying conditions

Seasonal influenza vaccination for persons with underlying medical conditions was recommended by 91% (43 of 47) of CA in 2008/09, increasing to 96% (51 of 53) in 2022/23 (Fig. 4). Recommendations covered a wide range of health conditions, including cardiovascular disease, chronic respiratory diseases (COPD and asthma), kidney or liver disease, diabetes, obesity, cancer, HIV/AIDS, hepatitis, long-term aspirin use, and immunosuppression, including chemotherapy. A similar proportion of CA reported vaccine coverage for this group in 2008/09 and 2022/23 (33% [14 of 43] versus 29% [15 of 51]), with reported coverage ranging from <1% to 72% and <1%–94% respectively.

Based on CA providing payment information, 82% (n = 32, 2021/22) to 84% (n = 31, 2020/21) reported that influenza vaccination for persons with chronic conditions was offered free of charge (Supplementary Fig. S5).

### Children

The proportion of CA including healthy children without chronic conditions in their seasonal influenza vaccination programme more than doubled, from 30% (14 of 47) in 2008/09 to 62% (33 of 53) in 2022/23 (Fig. 4). Age ranges varied widely (Supplementary Fig. S3). In 2022/23, 70% (23 of 33) offered vaccination to children from 6 months of age with varying upper age limits (up to 18 years). Among CA with recommendations, reporting of vaccination coverage increased from 29% (4 of 14) in 2008/09 to 67% (22 of 33) in 2022/23 (Fig. 4). The reported coverage ranged from <1% to 41% in 2008/09 and from <1% to 59% in 2022/23.

Influenza vaccination for children was provided free of charge in 65% (n = 17, 2021/22) and 72% (n = 23, 2022/23) of CA with available payment data between 2020/21 and 2022/23, representing the lowest proportion of free vaccination across the target groups (Supplementary Fig. S5).

### Other groups

In addition to the core target groups recommended for influenza vaccination by SAGE, several CA also reported recommendations for other target populations. Residents of long-term care facilities (LTCFs) were the most commonly included group with recommendations increasing from 36 of 47 CA (77%) in 2008/09 to 51 of 53 CA (96%) in 2022/23. The definition of LTCF varied and included settings such as institutions for older adults, orphanages, and boarding schools. Recommendations were also reported for a range of occupational and societal groups, including people working with animals (e.g., veterinarians, livestock handlers, husbandry workers), uniformed personnel (e.g., police, military, firefighters), educators, transportation workers, carers, and travellers. In 2008/09, 15 of 47 CA (32%) reported recommendations for one or more of these groups increasing to 32 of 53 (60%) in 2022/23. Only one CA reported migrants as a separate target group, and only for a single season. Further breakdown of recommendations by specific subgroups could not be determined because of inconsistent reporting across CA and seasons.

## Discussion

This report highlights considerable progress in seasonal influenza vaccination programmes in the WHO European Region over a 15-year period, in particular the expansion of target group recommendations and increased vaccine use in middle-income CA. Despite these advances, challenges persist, including low vaccination uptake, limited per-capita vaccine supplies in lower-resourced settings, and gaps in coverage monitoring.

By 2021/22, all CA had established a seasonal influenza vaccination programme, making WHO/Europe the first among the six WHO Regions to achieve this.^16^ In addition, since 2008/09, vaccine distribution in the Region more than doubled, an increase driven mainly by UMICs. Nonetheless, per-capita availability in LMICs remained substantially below that of HICs, where ten times as many doses were distributed in 2022/23, a finding consistent with global data documenting limited influenza vaccine use in many low- and middle-income countries.^20^

Encouragingly, since 2008/09, a growing number of CA have expanded their influenza vaccination programme to include additional target groups, particularly children and pregnant women, and most CA, regardless of income group, have adopted the global recommendations on priority populations. An increasing proportion of CA also reported recommendations for “other” groups beyond the five SAGE priority groups, including a range of occupational and societal groups, however, recommendations for population groups defined by socioeconomic, Indigenous, or migration status were rarely reported.

Despite this expansion in recommended target groups, critical gaps remain in monitoring vaccination uptake among target groups. While coverage monitoring among older adults is now performed by nearly all CA, fewer than one-third report coverage for persons with chronic conditions, and data for other target groups remain limited. Similar challenges exist for COVID-19 vaccination^21^ suggesting that monitoring gaps reflect broader structural constraints, including limitations in information systems for collecting data on adult vaccination across diverse target populations and delivery settings. These gaps also raise equity concerns. Socioeconomic disadvantage is associated with increased influenza risk and severity,^22^^,^^23^ however, without routine tracking of vaccination uptake across population groups, potential disparities in coverage cannot be identified or addressed. Systematic monitoring of coverage is essential for assessing programme performance and guiding targeted improvements and should be prioritized by health policy makers as an important area for investment.

Despite the broadening of recommendations and improvement in the number of vaccine doses procured, the limited data available in our study indicate that vaccination uptake continues to be low across target groups, except for a few CA. Compared with our previous report,^15^ there was only a modest increase in the number of CA reaching the 75% coverage goal for older adults. Furthermore, while reported coverage for older adults has increased in several CA since the beginning of the study period, large disparities in coverage persist across income groups with median CA-level coverage of 5% in LMICs compared with 55% among HICs in 2022/23. Notwithstanding these challenges, influenza vaccination coverage among older adults rose in many CA during the first winter of the COVID-19 pandemic (2020/21) and appeared to have been sustained in subsequent years. This increase may reflect heightened risk perception and demand during the COVID-19 pandemic, as well as changes in national vaccination programmes (e.g., expanded provision of vaccines free of charge and increased availability of vaccine doses). The increasing coverage among older adults during the pandemic contrasts with reports from other parts of the world where COVID-19 negatively affected seasonal influenza vaccination programmes,^24^ suggesting a degree of resilience in the national programmes in the WHO European Region. Comparable trend analyses, including post-COVID-19, in other target groups (e.g., health workers and adults with chronic conditions) were not possible due to incomplete and inconsistent multi-season (over-time) coverage reporting and changes in target group definitions over time.

The limited available data on vaccine formulation indicated a gradual shift from trivalent to quadrivalent vaccines over time, reflecting global guidance in 2013 to consider including both influenza B lineages in seasonal vaccine compositions.^25^ However, in September 2023, WHO updated its guidance stating that the inclusion of a B/Yamagata lineage antigen is no longer warranted.^26^ Accordingly, CA are expected to gradually return to trivalent formulations, a shift that could not be captured within our study period. Use of high-dose and adjuvanted vaccines was reported by both middle- and high-income CA, however, incomplete reporting and the lack of information on their proportion relative to standard vaccines prevent a meaningful assessment of the actual use across the Region and income settings. In addition, our data suggested that HICs predominantly use prefilled syringes while middle-income CA continue to use multidose vials. The higher upfront procurement cost and storage requirement of prefilled syringes^27^ may partly explain this difference, although prefilled syringes may offer operational advantages such as faster administration and reduced dose wastage and handling errors, which could outweigh the higher initial costs in some settings.^28^ More targeted studies are needed to better understand factors influencing vaccine choices, including financing, and cold chain and logistics capacity.

Barriers to influenza vaccination are well documented, especially in HICs, and include individual-level demand-side factors (e.g., concerns about vaccine safety and effectiveness, low perceived need, and lack of trust in healthcare systems), as well as access constraints (e.g., cost, inconvenience, and the need for annual vaccination).^29^ Although most CA reported free provision of influenza vaccines for several target groups, out-of-pocket payment remains common and is likely to pose a barrier for increasing vaccination uptake in the WHO European Region. In addition, uptake is influenced by programme and health systems characteristics, such as programme prioritization and implementation capacity, vaccine availability, national policies (e.g., mandatory vaccination for health workers), and delivery strategies (e.g., vaccination offered through primary care, pharmacies, schools, or workplaces), which likely contribute to the variations in coverage observed between CA and across target groups. Evidence suggests that reducing access barriers can improve uptake, for example lowering out-of-pocket costs has increased uptake among health workers and may narrow socio-economic disparities in coverage,^22^ and school-based delivery in paediatric programmes (including those using LAIV) have been associated with higher coverage.^30^ However, programme, policy and delivery characteristics, and the potential differences between CA, could not be assessed in this analysis as information was not collected. To help address these barriers, methodological guidance has recently been developed to systematically identify access and demand constraints and inform interventions to improve uptake.^31^

Beyond demand and affordability issues, vaccine availability itself remains an important constraint on achieving higher coverage in some settings. Vaccine procurement in many LMICs, including in the WHO European Region, is reported to be limited by funding capacity, high vaccine prices, competing health priorities, lack of long-term vaccination policies, as well as gaps in data on influenza burden and cost-effectiveness.^32^, ^33^, ^34^

Our analysis is subject to several limitations. First, vaccination coverage data were incomplete across target groups, with missing numerator and denominator data, and some sources may not be fully up-to-date, particularly for historical data. Consequently, presented trends over time reflect only a subset of CA with consistent reporting and may not be representative of the Region. Multi-season trend analysis was only feasible for older adults, as data for other target groups (e.g. health workers and persons with chronic conditions) were too incomplete to reliably assess trends, including post-COVID-19. Increases over time in the number of CA reporting coverage also limit comparability of median coverage estimates across seasons. Comparisons across target groups are further complicated by overlapping categories that are not mutually exclusive. For example, vaccination of persons aged 65 years or older with chronic conditions may be reported under the category ‘older adults’, ‘chronic underlying conditions’, or both. In addition, coverage for adults with chronic conditions is typically reported for all adults (≥18 years) without further age stratification (e.g., <60/65 years versus older), reflecting the structure of the JRF survey instrument. This limits assessment of uptake among younger at-risk adults, and underscores the importance of introducing more granular, age-stratified reporting options in future surveys.

Second, target group definitions and monitoring approaches vary across CA and change over time (including age thresholds, included medical conditions, and definition of health workers); therefore, trends and between-CA comparisons should be interpreted with caution. Standardisation of older-adult coverage to a common age threshold (e.g., ≥65 years) was not feasible due to incomplete reporting of age thresholds across seasons and an insufficient number of CA with consistent multi-season data using a ≥65-year definition. Formal breakpoint analyses (e.g., joinpoint or interrupted time series) were not conducted because changes in target group definitions, incomplete reporting, and limited comparability of coverage measures over time could create artificial changes in trends unrelated to true changes in uptake.

Third, vaccine dose distribution data were not consistently reported by a large proportion of CA, underestimating the actual availability of vaccines in the WHO European Region. Data may also exclude vaccines available through the private market. However, data were mainly missing from HICs, including those with known high procurement volumes; therefore, the observed per capita differences by income group are likely reliable.

Fourth, since this was an ecological analysis, we could not draw causal conclusions about vaccination uptake at the individual level. In addition, information on key determinants of vaccination uptake, including delivery settings, accessibility, and mandatory vaccination policies by target group, were not included in the surveys, further limiting our ability to examine and compare CA-level factors associated with vaccine uptake. Lastly, because payment data were only collected from 2020/21 onwards, we were unable to examine whether changes in policy (e.g., introduction or withdrawal of free vaccination) affected influenza vaccination coverage over time.

In conclusion, strengthening seasonal influenza vaccination in the WHO European Region remains a key priority for public health and health security and is central to equitable pandemic preparedness. Building on recent progress, national governments should continue to expand vaccination programmes, ensure that vaccines are provided free of charge and that access is convenient. Governments should also intensify efforts to reach underserved and hard-to-reach populations (including migrants and refugees, socioeconomically disadvantaged communities, remote/rural populations, and other marginalized groups) through strengthened community-based and outreach delivery strategies.

Sustained commitment to monitoring coverage and other performance indicators will be critical to tracking progress and guiding national and global policy development. Furthermore, documenting the burden of influenza and demonstrating the positive impact of vaccination programmes in terms of hospitalizations averted and lives saved or economic gains could help encourage CA to increase their vaccine supplies and coverage.

## Contributors

Margaux Marie Isabelle Meslé (MMIM) contributed to the study methodology and conceptualization, curated the data, conducted the data analysis, prepared the visualisations, and drafted the original manuscript. Marc-Alain Widdowson contributed to manuscript review and editing, acquired funding, and oversaw project administration. Pernille Jorgensen (PJ) conceptualized the study, curated the data, drafted and revised the manuscript, and provided supervision and project administration. MMIM and PJ directly accessed and verified the underlying data. All authors had final responsibility for the decision to submit for publication. The authors alone are responsible for the views presented in this manuscript and they do not necessarily reflect the views, decisions or policies of the World Health Organization.

## Data sharing statement

The data analysed in this study are publicly available. Historical influenza vaccination data up to 2014/15 are available from the WHO/Europe Gateway (https://gateway.euro.who.int/en/datasets/influenza/). More recent data were obtained from the WHO-UNICEF Joint Reporting Form on Immunization (eJRF) and are available at: https://immunizationdata.who.int/global/wiise-detail-page/influenza-vaccination-policy?ISO_3_CODE=&YEAR=. There are no restrictions to access. The analytical dataset generated during the current study is available from the corresponding author on reasonable request.

## Editor note

The Lancet Group takes a neutral position with respect to territorial claims in published maps and institutional affiliations.

## Declaration of interests

The authors declare no conflict of interest for this work.

## Appendix A Supplementary data

Supplementary Figs. S1–S5 and Tables S1 and S2

Title_tRu

## Footnotes

b Data for missing seasons were imputed as described under Methods.

c The designation employed and the presentation of this material do not imply the expression of any opinion whatsoever on the part of the Secretariat of the World Health Organization concerning the legal status of any country, territory, city or area or of its authorities, or concerning the delimitation of its frontiers and boundaries.

d Notes: The four United Kingdom nations (England, Scotland, Wales, and Northern Ireland) reported coverage separately in both seasons and are shown as separate data points. The horizontal dashed line represents the WHA target of 75% coverage.
