# Supplementary material for: Progress and challenges in seasonal influenza vaccination across 54 countries and areas in the WHO European Region, 2008/09–2022/23: a repeated cross-sectional ecological study
Source: Lancet Reg Health Eur. 2026 Apr 19;66:101681. doi: 10.1016/j.lanepe.2026.101681 (PMC13103574; doi:10.1016/j.lanepe.2026.101681)
Supplement: Supplementary Figs. S1–S5 and Tables S1 and S2 [file mmc1.docx]

Appendix to “Progress and challenges in seasonal influenza vaccination across 54 countries and areas in the WHO European Region, 2008/09-2022/23: a repeated cross-sectional ecological study”

Margaux MI Meslé, Marc-Alain Widdowson, and Pernille Jorgensen


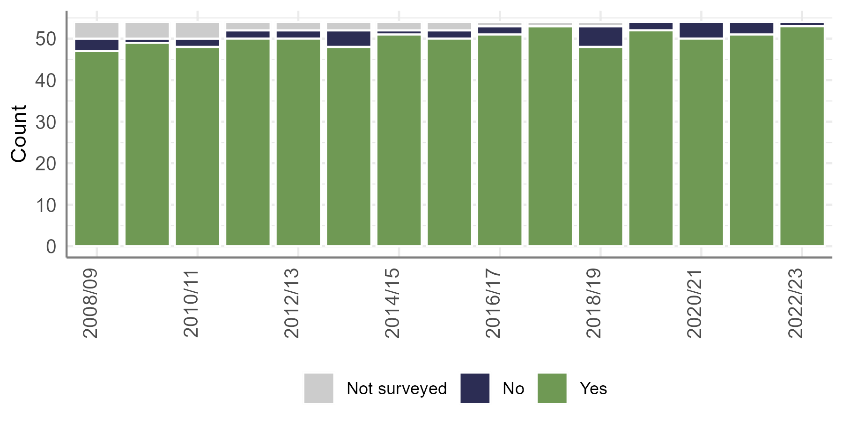


Supplementary Figure S1: Number of countries or areas in the WHO European Region included in, and responding to, annual surveys on seasonal influenza vaccination.

.

*Supplementary Table S1: Summary of survey indicators used to collect information on seasonal influenza vaccination in the WHO European Region.*

| Variable name | Description of variable | First season of collection | Target groups included |
| --- | --- | --- | --- |
| Coverage | Data relating to uptake: numerator and denominator, and coverage (%) | 2008/09 | Older adults, health workers, children, chronic underlying conditions, pregnant women, long term care facilities residents |
| Doses distributed | Number of seasonal vaccine doses distributed | 2008/09 | None - general population |
| Recommendations | Was seasonal influenza vaccination recommended? | 2008/09 | Older adults, health workers, children, chronic underlying conditions, pregnant women, long term care facilities residents |
| Governmental policy | Does the country or area have a governmental vaccination policy in place? | 2013/14 | None - general population |
| Vaccine types | What vaccines were used (trivalent, quadrivalent, Live Attenuated Influenza Virus, high dose, adjuvanted)? | 2015/16 | None - general population |
| Payment policy | Was payment required for influenza vaccines by different risk groups? | 2020/21 | Older adults, health workers, children, chronic underlying conditions, pregnant women, long term care facilities residents |
| Delivery programme | What type of programme was in place for delivery of vaccines | 2022/23 | Older adults, health workers, children, chronic underlying conditions, pregnant women, long term care facilities residents |

Supplementary Table S2: List of countries or areas reporting to the WHO Regional Office for Europe, by World Bank income group (2024 classification).

| World Bank income classification (2024) | Countries or Areas included |
| --- | --- |
| Lower middle income  (n=4) | Kyrgyzstan, Tajikistan, Ukraine and Uzbekistan |
| Upper middle income  (n=16) | Albania, Armenia, Azerbaijan, Belarus, Bosnia and Herzegovina, Bulgaria, Georgia, Kazakhstan, Montenegro, North Macedonia, Republic of Moldova, Russian Federation, Serbia, Turkmenistan, Türkiye, and Kosovo (in accordance with UN Security Council resolution 1244 (1999)) |
| High income  (n=34) | Andorra, Austria, Belgium, Croatia, Cyprus, Czechia, Denmark, Estonia, Finland, France, Germany, Greece, Hungary, Iceland, Ireland, Israel, Italy, Latvia, Lithuania, Luxembourg, Malta, Monaco, Netherlands, Norway, Poland, Portugal, Romania, San Marino, Slovakia, Slovenia, Spain, Sweden, Switzerland and United Kingdom of Great Britain and Northern Ireland |


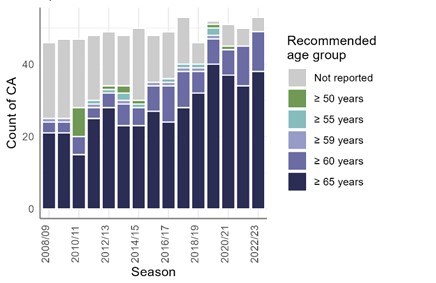


Supplementary Figure S2: Count of countries and areas reporting information on the recommended age groups for seasonal influenza vaccination for older adults in the WHO European Region.


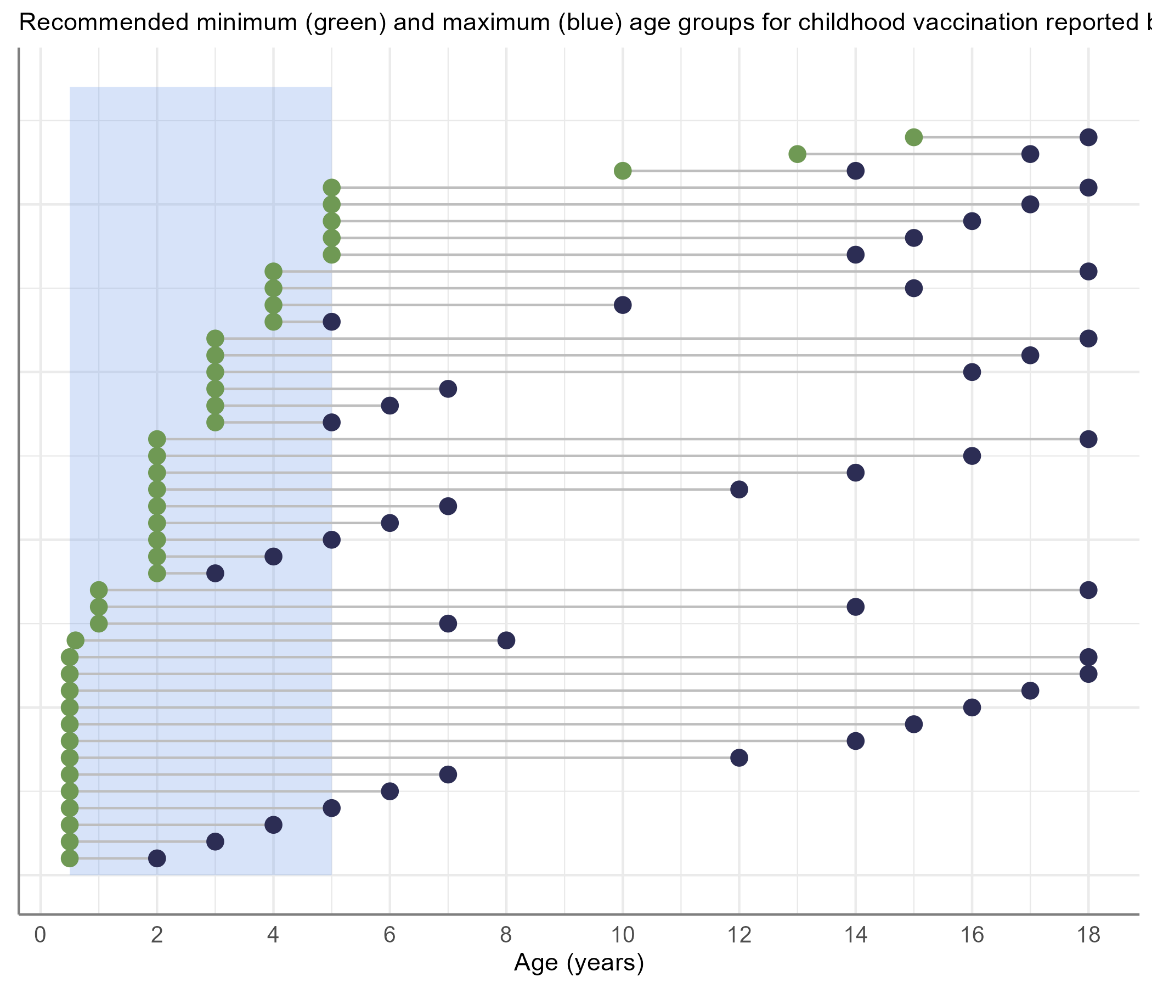


Supplementary Figure S3: Recommended age ranges for children for seasonal influenza vaccination, as reported by countries and areas over 15 seasons. Green dots indicate the minimum age and blue dots the maximum age. The shaded box indicates the age group recommended by SAGE. Markers and lines summarize reported age ranges across countries/areas and seasons and do not represent single country/area reports.

*Supplementary Figure S4: Sensitivity analysis of median influenza vaccination coverage among older adults by World Bank income group, comparing countries and areas with coverage data for all seasons (2008/09-2022/23; n=24) with those with data for ≥12 seasons (n=33).*


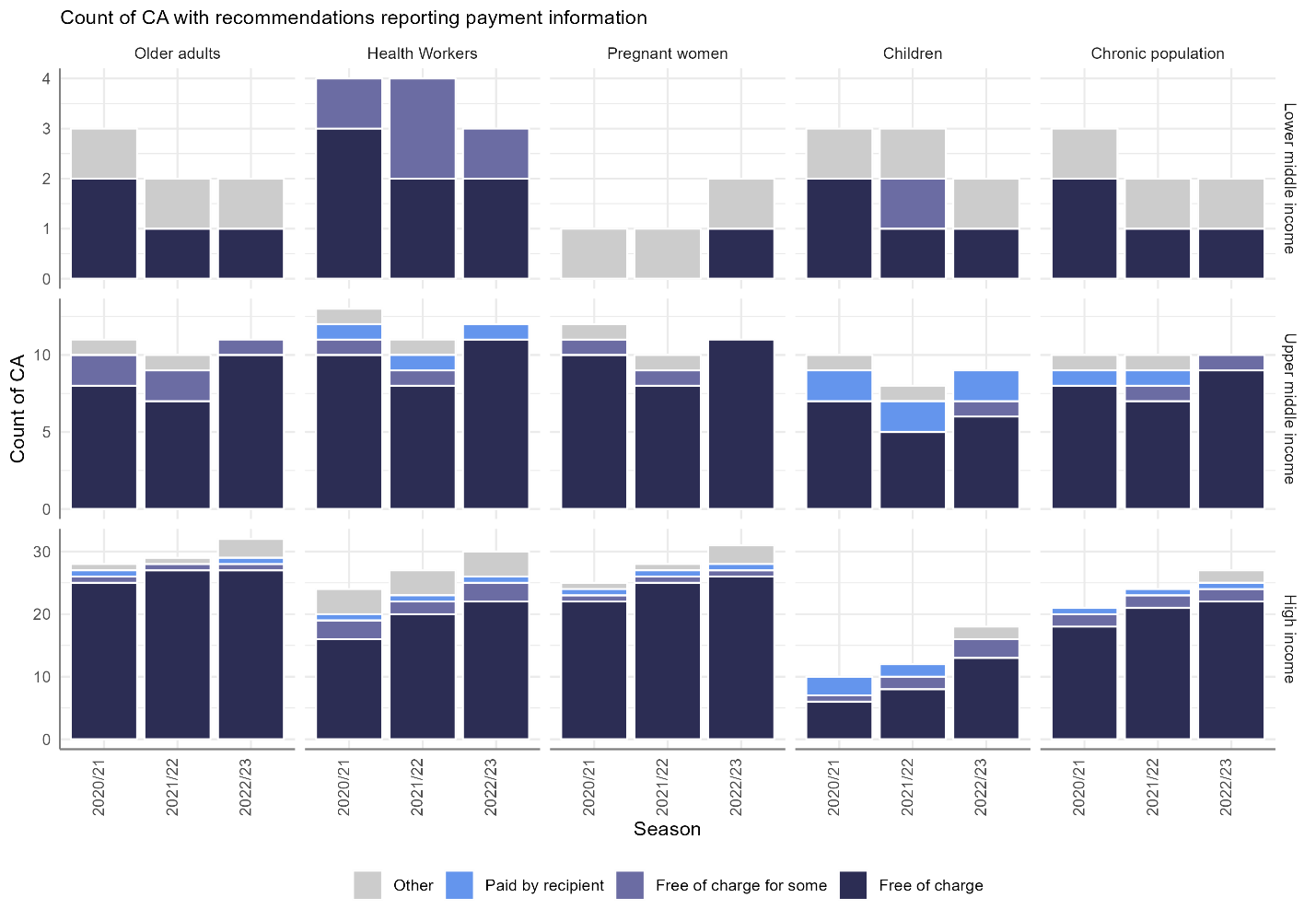


Supplementary Figure S5: Payment for seasonal influenza vaccination by target group, World Bank income group, and season, among countries and areas reporting payment information.
