# Supplementary material for: Progress and challenges in seasonal influenza vaccination across 54 countries and areas in the WHO European Region, 2008/09–2022/23: a repeated cross-sectional ecological study
Source: Lancet Reg Health Eur. 2026 Apr 19;66:101681. doi: 10.1016/j.lanepe.2026.101681 (PMC13103574; doi:10.1016/j.lanepe.2026.101681)
Supplement: Title_tRu [file mmc2.docx]

**Название:** Достижения и сложности в области вакцинации против сезонного гриппа в 54 странах и на территориях Европейского региона ВОЗ, 2008/09–2022/23 гг.: повторное перекрестное экологическое исследование

**Резюме**

**Справочная информация**
Сезонный грипп ежегодно вызывает до 5 млн тяжелых случаев заболевания и 650 тыс. летальных исходов. Группам высокого риска и работникам здравоохранения рекомендуется проходить вакцинацию, однако масштабы ее проведения во всем мире остаются недостаточными. В настоящем документе, содержащем рекомендации относительно дальнейших мер политики и инвестиций, анализируется ход развития программ вакцинации против сезонного гриппа в 54 странах и на территориях, входящих в состав Европейского региона ВОЗ, за период с 2008/09 по 2022/23 гг.

**Методология**

Авторы провели описательное повторное перекрестное экологическое исследование, использовав национальные данные, полученные с помощью единой формы отчетности об иммунизации ВОЗ-ЮНИСЕФ и извлеченные из докладов Европейского центра по борьбе с болезнями и их профилактике, с тем чтобы проанализировать дозировки и виды вакцин против гриппа, медицинские рекомендации, охват вакцинацией и схемы оплаты. Оценка тенденций, касающихся числа введенных доз вакцины на душу населения и охвата пожилого населения на протяжении 15 сезонов, проводилась в разбивке по группам дохода согласно классификации Всемирного банка.

**Выводы**

За период с 2008/09 гг. число введенных доз вакцины против гриппа увеличилось вдвое, причем наибольший рост продемонстрировали страны и территории с уровнем дохода выше среднего (в три раза) и ниже среднего (в восемь раз). Тем не менее по состоянию на 2022/23 гг. показатель доступности вакцин в дозах на душу населения в странах с доходом выше среднего оставался значительно более высоким, чем в странах с доходом ниже среднего (145,7 против 38,5, т. е. в четыре раза выше). Рекомендации в отношении вакцинации постепенно распространяются на новые целевые группы, однако система мониторинга охвата остается недостаточно эффективной: так, данные по ключевым группам, включая работников здравоохранения, предоставляют менее 50 % стран и территорий. В 2022/23 гг. медианный показатель охвата вакцинацией пожилых людей составил 55 %, а рекомендованного Всемирной ассамблеей здравоохранения целевого уровня в 75 % добились лишь четыре страны и территории.

**Интерпретация результатов**

Европейский регион ВОЗ добился значительных успехов в области вакцинации против гриппа: число стран и территорий, рекомендующих вакцинацию ключевым целевым группам, продолжает расти. В то же время охват приоритетных групп остается низким, а обеспеченность вакцинами значительно варьируется в зависимости от уровня дохода. Для дальнейшего увеличения охвата вакцинацией групп высокого риска необходимо продолжать инвестиции в национальные программы, особенно в странах и на территориях со средним уровнем дохода.

**Финансирование**

Данная работа была подготовлена благодаря финансовой поддержке партнеров Механизма обеспечения готовности к пандемическому гриппу.
